# Association between resistin and fibroblast growth factor 23 in patients with type 2 diabetes mellitus

**DOI:** 10.1038/s41598-018-32432-z

**Published:** 2018-09-18

**Authors:** Akio Nakashima, Keitaro Yokoyama, Daiji Kawanami, Ichiro Ohkido, Mitsuyoshi Urashima, Kazunori Utsunomiya, Takashi Yokoo

**Affiliations:** 1 Division of Nephrology and Hypertension, Department of Internal Medicine, Jikei University School of Medicine, Tokyo, Japan; 2 Division of Diabetes, Metabolism and Endocrinology, Department of Internal Medicine, Jikei University School of Medicine, Tokyo, Japan; 3 Division of Molecular Epidemiology, Jikei University School of Medicine, Tokyo, Japan

## Abstract

Fibroblast growth factor 23 (FGF23) is associated with cardiovascular disease and all-cause mortality in patients with diabetes mellitus. Insulin resistance has recently been reported to increase FGF23 levels, and resistin is a peptide that mainly regulates insulin resistance. However, few studies have investigated the association between FGF23 and resistin. A total of 422 patients with diabetes mellitus were recruited for this cross-sectional study to examine the association between resistin and intact FGF23. The mean ( ± standard deviation) age was 63.1 ± 11.9 years, and the median HbA1c was 6.7% (range, 6.1–7.1%). The mean estimated glomerular filtration rate (eGFR) was 66.2 ± 23.1 mL/min/m^2^. Multiple regression analysis for resistin showed that logFGF23 (coefficient (Coef): 1.551; standard error (SE): 0.739; *P* = 0.036), C-peptide (Coef: 0.798; SE: 0.229; *P* = 0.001), ghrelin (Coef: 1.061; SE: 0.332; *P* = 0.001), intact parathyroid hormone (Coef: 0.022; SE: 0.099; *P* = 0.030), and eGFR (Coef: −0.091; SE: 0.017; *P* < 0.001) were all significantly associated with the resistin level. These associations were modified in patients with higher age, lower body mass index, and higher vitamin D levels. These results suggest that resistin is positively associated with serum FGF23 levels.

## Introduction

Patients with diabetes mellitus (DM) have a higher mortality, and their main cause of death is cardiovascular disease (CVD). Although many factors increase the risk of CVD, mineral-bone disorder has recently been regarded as an important risk factor for CVD.

Fibroblast growth factor 23 (FGF23), which is an endocrine hormone produced and secreted by osteocytes, affects renal tubules, and is involved in vitamin D metabolism, regulates phosphorus levels^1,2^. Previous studies have reported higher FGF23 levels as a risk factor for death, end-stage renal disease, and CVD^2–4^.

In patients with chronic kidney disease (CKD), insulin resistance increases as renal function decreases. A previous study reported higher insulin resistance as a risk factor for CVD in dialysis patients^5^. In addition, insulin resistance is thought to play an important role in the development of vascular dysfunction^6^. A study conducted in a mouse model of insulin signaling inhibition using genetically FGF23-deficient animals showed that insulin resistance was mediated by a vitamin D signaling pathway^7,8^. Accordingly, insulin resistance in DM patients may inhibit the influx of phosphate and elevate serum phosphate levels, thus increasing serum FGF23 levels to normalize serum phosphate levels. A previous study reported that serum phosphorus levels are associated with insulin resistance and vascular stiffness in hypertensive patients^9^. Another study involving type 2 diabetic CKD patients also reported that a higher homeostatic model analysis-insulin resistance (HOMA-IR) group had higher phosphorus levels^10^. Although, the detailed mechanisms of phosphorus and insulin resistance are unknown, it is plausible that elevation of FGF23 by elevated phosphorus levels affects insulin resistance, and insulin’s effect to increase renal phosphorus reabsorption through NaPi-II cotransporter affects renal phosphorus handling^11^.

Resistin, which is mainly expressed in monocytes and macrophages in humans, is the key peptide associated with insulin resistance. A previous study reported that resistin decreased insulin-stimulated glucose uptake in adipose tissue and skeletal muscle cells^12^. Serum resistin levels are higher in patients with DM than in non-DM subjects^13^, and they are strongly associated with HOMA-IR^14,15^. In addition, concentrations of resistin increase with reduced renal function, as in patients with a reduced glomerular filtration rate (GFR)^16^. Leptin and ghrelin, hormones that affect insulin resistance, also increase with decreased kidney function and are reported to be associated with resistin^17^. However, few studies have investigated the relationships between FGF23 and glucose cytokines such as resistin in CKD patients with DM. In addition, few reports have analyzed insulin resistance and FGF23 while including leptin and ghrelin. Thus, the aim of the present study aim was to analyze the association between resistin and FGF23 in CKD patients with DM. There are two types of tests to measure FGF23, C-terminal FGF23 assay and intact FGF23 assay. In this study we used intact FGF23 enzyme-linked immunosorbent assay (ELISA) kit because intact FGF23 assay better captures the biologically functional FGF23 molecule^18^.

## Results

### Patients’ characteristics

A total of 422 outpatients with type 2 DM were analyzed, and their characteristics are shown according to resistin quartiles in Table 1.

**Table 1:** Patients’ characteristics.

Resistin (ng/mL)	Quartile 1 (<2.75)	Quartile 2 (2.75–4.82)	Quartile 3 (4.82–7.91)	Quartile 4 (>7.91)	*P*
Number	**102**	**104**	**102**	**114**	
Age (y)	62 ± 9	61 ± 12	65 ± 12	65 ± 13	0.007
Male (%)	73.5	67.3	79.4	68.4	0.191
BMI (kg/m^2^)	24 ± 4	24 ± 4	25 ± 4	25 ± 4	<0.001
Duration (years)	12 ± 11	11 ± 10	13 ± 10	14 ± 11	0.369
sBP (mmHg)	127 ± 12	125 ± 12	129 ± 12	130 ± 15	0.161
dBP (mmHg)	77 ± 9	77 ± 10	76 ± 9	74 ± 10	0.780
Creatinine (mg/dL)	0.8 ± 0.3	0.8 ± 0.2	0.9 ± 0.4	1.6 ± 1.7	<0.001
eGFR (mL/min/1.73 m^2^)	75 ± 18	73 ± 19	66 ± 19	52 ± 27	<0.001
Alkaline phosphatase (IU/mL)	209 (179–252)	206 (175–270)	229 (188–277)	230 (187–273)	0.390
HbA1c (%)	6.5 ± 0.8	6.7 ± 1.0	6.8 ± 1.1	6.7 ± 1.0	0.005
Hemoglobin (g/dL)	14.1 ± 1.4	14.1 ± 1.4	13.8 ± 1.5	13 ± 2.0	<0.001
Albumin (g/dL)	4.5 ± 0.3	4.5 ± 0.4	4.4 ± 0.4	4.2 ± 0.5	0.002
Phosphate (mg/dL)	3.4 ± 0.5	3.5 ± 0.7	3.4 ± 0.5	3.6 ± 0.7	<0.001
Calcium (mg/dL)	9.4 ± 0.4	9.4 ± 0.4	9.3 ± 0.4	9.2 ± 0.5	0.003
IL-6 (g/mL)	7.5 (5.7–8.6)	7.8 (6.1–13.1)	8.0 (6.1–11.9)	8.4 (6.4–13.1)	<0.001
Fibroblast growth factor 23 (pg/mL)	69.1 (58.5–88.9)	66.8 (55.9–82.9)	76.6 (59.1–95.0)	85.1 (68.3–112.9)	<0.001
25(OH)D (ng/mL)	26.9 ± 11.2	24.5 ± 8.9	26 ± 8.1	23 ± 8.8	0.006
1,25(OH)_2_D (pg/mL)	55 ± 19.7	53.5 ± 16.2	50.2 ± 17.1	42.5 ± 19.0	0.167
iPTH (pg/mL)	36 (28–44)	36 (28–47)	38 (30–51)	50 (36–82)	<0.001
Resistin (ng/mL)	2.10 (1.37–2.48)	3.91 (3.18–4.35)	6.37 (5.41–7.18)	11.4 (9.36–16.5)	<0.001
C-peptide (ng/mL)	1.05 (0.59–1.59)	0.98 (0.55–1.63)	1.10 (0.74–1.68)	1.32 (0.69–2.50)	0.238
Ghrelin (ng/mL)	0.47 (0.23–0.82)	0.47 (0.28–1.04)	0.51 (0.32–0.88)	0.87 (0.47–1.50)	0.001
Leptin (ng/mL)	3.32 (1.83–5.81)	4.13 (1.80–7.87)	3.57 (1.68–7.34)	4.10 (1.82–11.05)	0.125
Insulin secretion (%)	16.8	12.5	14.7	22.8	0.201
Sulfonylurea (%)	11.8	11.5	8.8	7.9	0.717
Dipeptidyl peptidase-4 inhibitor (%)	23.5	29.8	20.6	15.8	0.092
Metformin (%)	44.1	38.5	29.4	15.8	0.017
Alpha-glucosidase inhibitor (%)	15.7	13.5	12.7	14.1	0.940
Insulin therapy (%)	23.5	29.8	34.3	42.1	0.029

Abbreviations: BMI, body mass index; sBP, systolic blood pressure; dBP, diastolic blood pressure; eGFR, estimated glomerular filtration rate; HbA1c, hemoglobin A1c; Il-6, interleukin-6; 25OHD, 25 hydroxyvitamin D; 1,25(OH)_2_D, 1,25 dihydroxyvitamin D; iPTH, intact parathyroid hormone.

The mean (±standard deviation) age was 63.1 ± 11.9 years, the median HbA1c level was 6.7% (range, 6.1–7.1%), and the median duration of DM was 10 years (range, 4–20 years). Males accounted for 72.1% of subjects, and insulin therapy was prescribed for 32.7%. Patients with higher resistin levels were older and had higher creatinine levels than those with lower resistin levels (Table 1). In addition, patients with higher resistin levels had lower levels of 25(OH)D, hemoglobin, and calcium, and higher PTH and phosphorus levels. However, no significant differences in duration of DM, blood pressure, or 1,25(OH)_2_D levels were seen between resistin groups. Ghrelin increased with the resistin level, but no associations were detected for C-peptide or leptin. Intact FGF23 was analyzed using an ELISA kit (Kainos Laboratories, Tokyo, Japan). Serum FGF23 levels were found to increase with decreased kidney function.

### Multiple regression analysis for resistin

**Figure 1:**
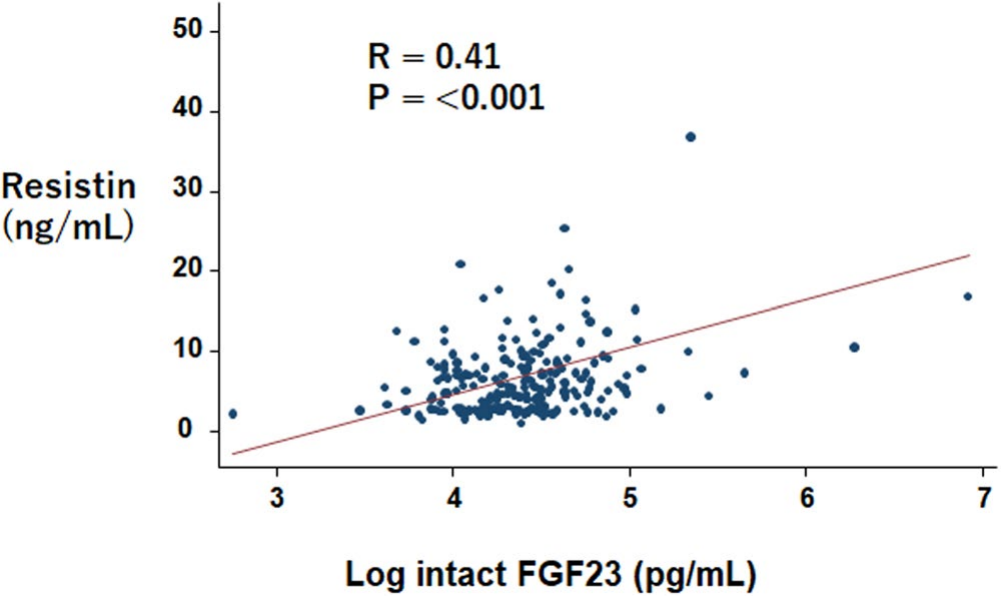
Scatter plots and correlation coefficients between FGF23 and resistin Abbreviation: FGF23, fibroblast growth factor 23.

The correlation coefficient for resistin and FGF23 was 0.41 (*P* < 0.001; Fig. 1). Table 2 presents the results of the multiple regression analysis for resistin with patients’ characteristics, markers of bone mineral metabolism, and diabetes markers. The following were significantly associated with resistin levels: logFGF23 [coefficient (Coef): 1.551; standard error (SE): 0.739; *P* = 0.036), C-peptide (Coef: 0.798; SE: 0.229; *P* = 0.001), ghrelin (Coef: 1.061; SE: 0.332; *P* = 0.001), intact parathyroid hormone (Coef: 0.022; SE: 0.099; *P* = 0.030), and eGFR (Coef: −0.091; SE: 0.017; *P* < 0.001).

**Table 2:** Multiple regression analysis for resistin.

	Coef.	SE	*P*-value
LogFGF-23	1.551	0.739	0.036
C-peptide	0.798	0.229	0.001
Ghrelin	1.061	0.332	0.001
Leptin	−0.035	0.040	0.390
Age	−0.042	0.025	0.097
25OHD	−0.025	0.031	0.423
iPTH	0.022	0.099	0.030
eGFR	−0.091	0.017	<0.001
Body mass index	0.002	0.029	0.943
IL-6	0.091	0.061	0.136
HbA1c	0.323	0.282	0.254

Abbreviations: FGF-23, fibroblast growth factor 23; 25OHD, 25 hydroxyvitamin D; iPTH, intact parathyroid hormone; eGFR, estimated glomerular filtration rate; Il-6, interleukin-6; HbA1c, hemoglobin A1c.

### Multiple regression analysis for resistin categorized by age, vitamin D status, and body mass index (BMI)

In younger patients (<64 years old), resistin was not associated with the FGF23 concentration. However, in older patients (≥64 years), resistin increased proportionally with the FGF23 level (Table 3; P for interaction, 0.015). Patients were also categorized by 25OHD status, and individuals with low vitamin D (<24 ng/mL) showed no significant association between resistin and FGF23 (Table 3; P for interaction, 0.034). In addition, among patients with a low BMI (<23.9 kg/m^2^), resistin was significantly associated with the FGF23 level (Table 3; P for interaction, 0.893).

**Table 3:** Multiple regression analysis of logFGF23 to resistin categorized by age, vitamin D status, and body mass index.

		Number	Coef.	SE	*P*-value
Age (y)	<64	197 (46.7%)	−0.243	1.065	0.819
	≥64	225 (53.3%)	2.341	1.023	0.023
25-hydroxyvitamin D (ng/ml)	<24	195 (46.2%)	−0.898	1.195	0.454
	≥24	227 (53.8%)	3.471	0.941	<0.001
Body mass index (kg/m^2^)	<23.9	210 (50.0%)	2.895	1.042	0.006
	≥23.9	212 (50.5%)	−0.417	1.104	0.706

## Discussion

In this cross-sectional study of patients with type 2 DM, serum resistin was positively associated with serum FGF23 levels after adjusting for known variables that affect the FGF23 level. The association between resistin and FGF23 varied by age, 25OHD, and BMI. These findings suggest that resistin is associated with FGF23 levels, and insulin resistance may be a key factor in CKD bone-mineral metabolism. This study is the first to show that resistin and FGF23 are significantly associated, even on multiple regression analysis including renal function, PTH, vitamin D status, and markers of diabetes.

Resistin is mainly secreted by adipocytes in mice, where this adipokine is the key peptide associated with insulin resistance, hyperglycemia, and obesity^19,20^. On the other hand, in humans, this peptide is expressed mainly by macrophages and is involved in inflammatory processes^21^. Previous studies have reported that resistin is associated with obesity, visceral fat, and the etiology of DM^22,23^. In the present study, serum resistin levels were inversely correlated with eGFR after adjustment on multiple regression analysis. Consistent with the present observation, previous studies have also reported that resistin is inversely associated with kidney function^16,24^.

A novel finding of the present study was that FGF23 was associated with resistin. This association was significant even after adjusting for eGFR, PTH, 25OHD, 1,25(OH)_2_D, phosphorus, and interleukin (IL)−6. A previous study reported that FGF23 was associated with insulin resistance, obesity, hyperlipidemia, and visceral adiposity^25^. However, that study did not measure vitamin D status, and the present study is the first to show an association between resistin and FGF23, taking into account vitamin D status. Data on FGF23 and insulin resistance from animal models are ambiguous. In a mouse model, FGF23-deficient mice were hypoglycemic and showed increased peripheral insulin sensitivity and improved subcutaneous glucose tolerance^26^. In addition, another study reported that patients with higher FGF23 concentrations had higher mean concentrations of resistin^27^.

Several possible mechanisms may contribute to the association between resistin and FGF23. First, inflammatory cytokines such as C-reactive protein (CRP) and IL-6 are increased with FGF23 elevation^28,29^. Although resistin is regarded as an adipocytokine, previous studies have reported that resistin also increases concentrations of inflammatory cytokines^30,31^. Furthermore, resistin has been shown to have inflammatory properties^32^. With this background, resistin and FGF23 may form a positive feedback loop by which serum resistin levels increase in diabetic patients with impaired renal function. Investigation of the mechanisms by which these proteins upregulate each other is likely to prove interesting. In the present study, the association between resistin and FGF23 was significant even after adjusting for IL-6. However, levels of other inflammatory markers, such as CRP and tumor necrosis factor (TNF)-α, were not measured. A positive association between inflammatory cytokines and resistin thus cannot be excluded. Some reports have indicated that serum resistin levels correlate with levels of inflammatory markers such as IL-6 and TNF-α^33^. In the present study, resistin was associated with eGFR, but not with IL-6. However, the possibility that inflammatory mechanisms are involved in increased serum resistin levels among individuals with impaired renal function cannot yet be excluded. Future studies are required to clarify the relationship between serum resistin levels and inflammatory markers other than IL-6, such as CRP and TNF-α. Second, vitamin D affects the association between resistin and FGF23. A previous study reported that vitamin D deficiency exacerbated nonalcoholic fatty liver disease through Toll-Like receptor (TLR)-activation, possibly by way of endotoxin exposure in a wild-type rat model^34^. In patients with CKD, vitamin D deficiency is associated with insulin resistance^35^. In addition, activated vitamin D therapy has been shown to improve insulin resistance in patients on dialysis^36^. In the present study, no association between resistin and FGF23 was observed in groups with low vitamin D. As shown in Table 3, the association between FGF23 and resistin appears to be affected by vitamin D status, as well as by age and BMI. These results also indicate that the association between resistin and FGF23 is relatively weak in patients with higher insulin resistance.

The present study confirmed the association between resistin and FGF23 in groups with lower BMI, higher age, or higher vitamin D levels. Patients with higher BMI or lower vitamin D levels are reported to show greater insulin resistance^27^. In the present population, other mechanisms that increase insulin resistance (e.g., inflammation, klotho, malnutrition, etc.) may have decreased the association between resistin and FGF23. In addition, dietary consumption, information about exercise, and daily sunlight exposure were not checked, and these factors may have affected the study results.

Leptin is a peptide hormone produced and secreted by white adipose tissue. Leptin increases with decreased renal function and is related to obesity and insulin resistance. In addition, one study reported that leptin and resistin are related in CKD patients^37^. A recent study also showed that leptin is significantly associated with FGF23 among patients on hemodialysis^38^. Although the details for the mechanism of association between leptin and FGF23 have yet to be elucidated, one study showed that leptin directly affects FGF23 synthesis in bone cells in the oblob mouse^39^. Ghrelin is a growth hormone-releasing peptide that regulates appetite, carbohydrate utilization, and insulin resistance. Ghrelin levels decrease with increasing renal function and are associated with mortality in CKD patients^37^. In the present study, the association between resistin and FGF23 remained significant even after adjusting for leptin and ghrelin. Leptin and ghrelin thus may not affect the association between resistin and FGF23.

Klotho is a peptide that affects the parathyroid gland and kidney via FGF23. Recently, klotho has been reported to affect insulin resistance^40^. Inhibition of tyrosine phosphorylation on insulin receptors and enhancement of glucose-induced insulin secretion through transient receptor potential V2 (TRPV2) are regarded as the mechanisms underlying the association between klotho and insulin resistance^41^. The association between FGF23 and resistin could potentially be through klotho.

## Limitations

The present study had several limitations. First, because the study design was cross-sectional, no cause-and-effect relationships could be established. Thus, one cannot determine whether increased resistin and FGF23 levels contribute to CKD or are a consequence of exacerbated CKD in diabetic patients. In addition, although eGFR was included as an index of kidney function on multivariate analysis for resistin, it could not completely account for the effect of kidney function. Thus, there is the possibility that the effect of kidney function may have remained in the multivariate analysis. Second, levels of other inflammatory markers, such as CRP and TNF-α, were not measured. Previous studies reported that CRP and TNF-α were associated with resistin concentrations. Although IL-6 was measured as an inflammatory cytokine and showed no significant association, other inflammatory cytokines may affect resistin levels. Third, interaction tests should be used with caution in data analyses. Most studies do not have sufficient power to detect such interaction effects, and the results of such tests are always exploratory in nature^42^. Fourth, although eGFR was included as a potential confounder on multivariate analysis, renal function was a strong confounder in this study, and its effect may not have been completely removed. Further study will be needed to investigate the association between FGF23 and resistin in patients with normal kidney function or on dialysis. Fifth, this study did not measure soluble klotho. Because klotho is reportedly associated with insulin resistance, the association between FGF23 and resistin could potentially result from the effects of klotho. Sixth, this study used an assay for intact FGF23, not C-terminal FGF23. There are two types of FGF23 ELISA assay, intact FGF23 and C-terminal FGF23. A previous study reported that intact FGF23 ELISA better captures the biologically functional FGF23 molecule^18^. The intact FGF23 assay measures the levels of full-length FGF23, while the C-terminal assay measures the levels of both full-length FGF23 and the C-terminal fragment. Previous studies reported that the full-length intact FGF23 protein is regarded as the biologically active form of the FGF23 hormone. On the other hand, the C-terminal fragment is generally regarded as inactive and does not affect the diuretic effect of phosphorus, and there is some evidence to suggest that C-terminal fragments may have anti-phosphaturic effects in mice^43^ or, conversely, phosphaturic activity in rats^44^. Future studies thus need to include both C-terminal FGF23 and intact FGF23 assays to clarify the association between resistin and FGF23.

In conclusion, the present study showed that resistin was associated with renal function, and that resistin was associated with serum FGF23 levels in type 2 DM patients. However, the underlying mechanisms have yet to be clarified. Future large-scale clinical studies are needed, and interventional studies are also required to elucidate the association between glucose metabolism and CKD mineral-bone metabolism.

## Materials and Methods

### Study design

This cross-sectional study was carried out as a collaboration among the Division of Nephrology and Hypertension, the Division of Diabetes, Metabolism and Endocrinology, and the Division of Molecular Epidemiology at Jikei University School of Medicine. The study protocol was reviewed and approved by the ethics committee of the Jikei Institutional Review Board, Jikei University School of Medicine (22–182). The accrual period was from April 2011 to March 2012. All patients provided written, informed consent prior to enrollment. The study was conducted in accordance with the 1975 Declaration of Helsinki, as revised in 2000. Outpatients at Jikei University Hospital were recruited.

### Study population and eligibility

Patients between 20 and 80 years old with type 2 DM, as diagnosed by physicians based on the Japanese diagnostic criteria in the Division of Diabetes, Metabolism and Endocrinology or the Division of Nephrology and Hypertension at Jikei University Hospital, were eligible and asked to participate in the study by the physicians. Participants had stable metabolic control of diabetes with HbA1c less than 9% in the recruitment period. Because patients with uncontrolled DM may have diabetic ketoacidosis, such patients were excluded. Patients with primary hyperparathyroidism, liver damage, active infections, dementia, active infectious disease, using active vitamin D, on dialysis, or who had received a kidney transplant were likewise excluded.

### Clinical evaluation

Disease duration (years) was defined as the interval between the diagnosis of diabetes and the clinical evaluation for entry into the study. Age, sex, height, weight, and blood pressure, as well as laboratory data, including concentrations of peripheral blood calcium (Ca) (normal range: 8.5–10.4 mg/dL), phosphate (P) (normal range: 2.5–4.5 mg/dL), and intact parathyroid hormone (iPTH) (normal range: 10–65 pg/mL), were evaluated. The eGFR was calculated according to the following Japanese standard formula based on inulin clearance: 194 × creatinine^−1.094^ × age ^−0.287^ (if female, ×0.739)^45^. CKD stages were defined based on eGFR levels as follows: Stage 1 CKD, eGFR ≥90 mL/min/1.73 m^2^; Stage 2 CKD, eGFR ≥60 to <90 mL/min/1.73 m^2^; Stage 3 CKD, eGFR ≥30 to <60 mL/min/1.73 m^2^; Stage 4 CKD, eGFR ≥15 to <30 mL/min/1.73 m^2^; and Stage 5 CKD, eGFR <15 mL/min/1.73 m^2^.

### Samples and 25OHD/1,25OHD/FGF23 measurements

Blood samples were collected at study entry. The blood was centrifuged for 10 minutes and divided into tubes kept at −80 °C until analysis. Serum levels of 25 hydroxyvitamin D (OHD) (ng/mL) and 1,25 dihydroxyvitamin D (1,25(OH)_2_D) (pg/mL) were measured at SRL Inc. (Hachioji, Tokyo, Japan), as described previously. Serum intact FGF23 was measured using a commercial ELISA kit (FGF23 Elisa kit, Kainos Laboratories, Tokyo, Japan). Intra- and interassay coefficients of variation were 10% and 14%, respectively^46^.

### Glucose cytokine measurements

Previous studies have reported that serum leptin and ghrelin levels increase with decreasing renal function^37,47^. Resistin and insulin resistance are also affected by leptin and ghrelin. In addition, leptin and ghrelin reportedly affect bone metabolism and are associated with bone mineral density. To exclude the effects of leptin and ghrelin, serum leptin and ghrelin levels were therefore measured and included as covariates. About 50 μL of serum from frozen samples were used to analyze resistin, leptin, and ghrelin levels. A Bio-plex suspension, bead-based, multiplexed array with a human diabetes assay panel (Bio-Rad Laboratories, Hercules, CA) was used to quantify serum glucose cytokines according to the manufacturer’s instructions. This multiplex system is a magnetic bead-based, multiple immunoassay. Intra-assay coefficients of variation were 4.0% for C-peptide, 3.0% for leptin, 4.0% for resistin, and 2.0% for ghrelin.

### Statistical analysis

Participants were divided into four categories by resistin concentration. Associations between CKD stage and patients’ characteristics were evaluated using analysis of variance, the Kruskal-Wallis test, and the chi-squared test, as appropriate. Hardy-Weinberg equilibrium was assessed by the chi-squared test. Correlation coefficients were calculated for resistin and FGF23. Multiple regression analysis was performed for resistin. The confounders of FGF23, C-peptide, ghrelin, leptin, age, 1,25(OH)_2_D, 25OHD, albumin, calcium, P, iPTH, eGFR, sex, BMI, IL-6, and HbA1c were considered, since a previous study reported that the interaction between insulin resistance and FGF23 was modified by BMI and age. In addition, mouse models have shown that the vitamin D signaling pathway affects insulin resistance in FGF23-deficient mice^7^. For these reasons, analyses of FGF23 and resistin were adjusted using these factors. Multiple regression analysis was conducted for resistin, with categories of age, vitamin D status, and BMI classified using the respective medians as cut-off values. Two-sided P-values less than 0.05 were considered significant. All statistical analyses were performed using STATA version 14.0 software (STATA Corp., College Station, TX).
